# Can restoration of heart rate in ESRD lower BNP? A case report

**DOI:** 10.1080/0886022X.2021.2003206

**Published:** 2021-11-18

**Authors:** Mahmoud M. Mohamed, Joel Raja, Atif Ibrahim, Hafiz Raza, Barry Wall, Mihaly Benjamin Tapolyai

**Affiliations:** a North Mississippi Medical Center, Tupelo, MS, USA; b The University of Tennessee Health Science Center, Memphis, TN, USA; c Memphis VA Medical Center, Memphis, TN, USA

Dear Editor,

We are pleased to present a case of End Stage Renal Disease (ESRD) on hemodialysis (HD) patient presenting with a complete heart block with an elevated B-type natriuretic peptide (BNP) from his baseline and review whether correcting the arrhythmia resulted in correction of his BNP. This is a 98-year-old gentleman with a past medical history of ESRD, who presented to the emergency department for shortness of breath and a decrease in heart rate. The patient went for his regular primary care provider visit where he was noted to be bradycardic with a heart rate in the 40 s. The patient’s significant vital signs were heart rate (HR) of 48 and blood pressure of 138/52 mm Hg. BNP was 2667 pg/mL. The electrocardiogram (ECG) revealed a complete heart block. His chest x-ray did not reveal any acute cardiopulmonary abnormalities. Cardiology service was consulted. Cardiac pacing pads were placed with the patient being promptly admitted to the Intensive Care Unit (ICU) with plans for permanent pacemaker placement. In the ICU, he remained hemodynamically stable with HR in the 30 s-40s while awaiting pacemaker placement. The patient had a dual-chamber pacemaker placement a day later. His symptoms of dyspnea resolved after the procedure. He was then transferred to a regular medical ward. The BNP was repeated 90 min after the procedure, which was still elevated at 2506 pg/ml. The patient then had his hemodialysis session with adequate ultrafiltration to reduce his body weight to his estimated dry weight, which brought down the BNP to 626 pg/mL. A repeat BNP after two more of his dialysis sessions was reduced to 409 pg/mL. The patient was discharged to a skilled nursing facility for rehabilitation.

In our case report, restoration of heart rate failed to lower BNP after 90 min in an anuric ESRD patient. BNP clearance from the human circulation is described to have short and long half-life components of 3.9 and 20.7 min, respectively [1]. More than four half-lives had been passed before rechecking the BNP value which should permit about 94–97% of BNP elimination [2].

The two types of natriuretic peptides namely, the brain natriuretic peptide (BNP) and N-terminal pro-B-type natriuretic peptide (NT-proBNP) are important markers in assessing heart pump function. They have a high negative predictive value rendering them particularly helpful to exclude heart failure [3] as a prognosticator. There is a strong correlation between BNP values and bioimpedance-measured volume status in ESRD patients [4]. Studies showed that BNP and NT-proBNP cleared by dialysis but still correlate with volume overload [5]. Natriuretic peptides are currently being studied for their role in the context of permanent cardiac pacemakers. Different pacemaker modes impact the hemodynamics differently, however, little is known about how BNP levels change when the cardiac rhythm is restored and presumably the cardiac output is improved. There is emerging data that the BNP-directed fluid management in ESRD may improve patient care [6]. Despite the scarcity of large studies done on this subject, some individual cases and small studies are reported over the years that were reviewed and are presented here.

In the physiologic mode, the atrio-ventricular delay seems to be affecting the BNP level remarkably which was studied in patients with sick sinus syndrome with organ versus non-organ heart disease [7]. Wu et al. followed a group of patients (*n* = 56) with permanent cardiac pacemakers in the medium to long term to assess the limitation of daily activity based on the levels of BNP. Their study showed that BNP levels in right ventricular apical pacing were notably higher compared to atrial pacing, although failed to show any difference in non-physiologic versus physiologic modes [8]. J Kojuri et al. performed a study on 79 patients with a mean age of 65 ± 13 analyzed the impact of pacing mode on BNP using 3 types of pacemaker (Dual-chamber, rate-modulated pacing (DDDR), single chamber pacing with dual chamber sensing (VDDR), Single chamber (VVIR)) method and concluded that patients with DDDR and VDDR pacing had lower brain natriuretic peptide levels than those with VVIR pacing (*p* < 0.0001) [9]. However, none of these studies included anuric renal failure patients; we speculate that perhaps the improved cardiac output was the factor that lowered renal perfusion and thus intravascular volume in the studies cited above. On contrary, higher concentrations of N-terminal, pro–B-type natriuretic peptide were observed in patients with Heart Failure with Preserved Ejection Fraction having Pacemaker Implantation compared with those without a pacemaker [10].

Our patient, however, gave us the unique opportunity to differentiate between the effects on BNP of an improved cardiac output, with improved cardiac rhythm, versus intravascular volume. This case demonstrates that this anuric patient’s rhythm restoration was not sufficient to lower BNP values yet ultrafiltration did. Thus, we seem to be able to confirm that it is solely the volume status that affects the BNP value, not the cardiac rhythm or cardiac output.

It is worth noting that none of the above-cited studies had patients with chronic kidney disease or end-stage renal disease. We concluded that restoration of heart rate failed to lower BNP in an anuric ESRD patient. BNP can be used as a marker of increased volume within the LV cavity in patients with complete heart block. More multi-center studies with long-term follow-up are needed to address the validity and reliability of BNP-directed fluid management in improving outcome of hemodialysis population.

**Table 1.** BNP on presentation, after procedure, and after dialysis.

	Presentation	After pacemaker placement	After dialysis
BNP pg/ml	2667	2506	626

This material has not been published previously, in whole or part, and is not under consideration for publication elsewhere. All authors had participated in the preparation of this manuscript, fulfilled criteria for authorship and have approved the paper in the current format. The authors have no conflict of interest to declare and are able to sign a formal Disclosure Statements and Copyright Transfer Forms for the paper.
